# Initiation of antiretroviral therapy before detection of colonic infiltration by HIV reduces viral reservoirs, inflammation and immune activation

**DOI:** 10.7448/IAS.19.1.21163

**Published:** 2016-09-15

**Authors:** Trevor A Crowell, James LK Fletcher, Irini Sereti, Suteeraporn Pinyakorn, Robin Dewar, Shelly J Krebs, Nitiya Chomchey, Rungsun Rerknimitr, Alexandra Schuetz, Nelson L Michael, Nittaya Phanuphak, Nicolas Chomont, Jintanat Ananworanich, for the RV254/SEARCH010 Study Group

**Affiliations:** 1 US Military HIV Research Program, Walter Reed Army Institute of Research, Silver Spring, MD, USA; 2 Henry M. Jackson Foundation for the Advancement of Military Medicine, Bethesda, MD, USA; 3 SEARCH, Thai Red Cross AIDS Research Centre, Bangkok, Thailand; 4 National Institute of Allergy and Infectious Diseases (NIAID), National Institutes of Health (NIH), Bethesda, MD, USA; 5 Virus Isolation and Serological Lab, National Cancer Institute at Frederick, Frederick, MD, USA; 6 Department of Medicine, Faculty of Medicine, Chulalongkorn University, Bangkok, Thailand; 7 Department of Retrovirology, Armed Forces Research Institute of Medical Sciences – United States Component, Bangkok, Thailand; 8 Department of Microbiology, Infectiology and Immunology, Faculty of Medicine, Université de Montréal, Montréal, QC, Canada; 9 Centre de Recherche du CHUM, Montreal, QC, Canada

**Keywords:** HIV, inflammation, CD4 lymphocyte count, highly active antiretroviral therapy, virus latency, infectious disease reservoirs

## Abstract

**Introduction:**

Colonic infiltration by HIV occurs soon after infection, establishing a persistent viral reservoir and a barrier to cure. We investigated virologic and immunologic correlates of detectable colonic HIV RNA during acute HIV infection (AHI) and their response to antiretroviral treatment (ART).

**Methods:**

From 49,458 samples screened for HIV, 74 participants were enrolled during AHI and 41 consented to optional sigmoidoscopy, HIV RNA was categorized as detectable (≥50 copies/mg) or undetectable in homogenized colon biopsy specimens. Biomarkers and HIV burden in blood, colon and cerebrospinal fluid were compared between groups and after 24 weeks of ART.

**Results:**

Colonic HIV RNA was detectable in 31 participants (76%) and was associated with longer duration since HIV exposure (median 16 vs. 11 days, *p=*0.02), higher median plasma levels of cytokines and inflammatory markers (CXCL10 476 vs. 148 pg/mL, *p=*0.02; TNF-RII 1036 vs. 649 pg/mL, *p<*0.01; neopterin 2405 vs. 1368 pg/mL, *p=*0.01) and higher levels of CD8+ T cell activation in the blood (human leukocyte antigen - antigen D related (HLA-DR)/CD38 expression 14.4% vs. 7.6%, *p* <0.01) and colon (8.9% vs. 4.5%, *p=*0.01). After 24 weeks of ART, participants with baseline detectable colonic HIV RNA demonstrated persistent elevations in total HIV DNA in colonic mucosal mononuclear cells (CMMCs) (median 61 vs. 0 copies/10^6^ CMMCs, *p=*0.03) and a trend towards higher total HIV DNA in peripheral blood mononuclear cells (PBMC) (41 vs. 1.5 copies/10^6^ PBMCs, *p=*0.06). There were no persistent differences in immune activation and inflammation.

**Conclusions:**

The presence of detectable colonic HIV RNA at the time of ART initiation during AHI is associated with higher levels of proviral DNA after 24 weeks of treatment. Seeding of HIV in the gut may have long-lasting effects on the size of persistent viral reservoirs and may represent an important therapeutic target in eradication strategies.

## Introduction

The gut-associated lymphoid tissue (GALT) is one of the first sites infiltrated by HIV during acute infection [1,2] and represents a major reservoir of latently infected cells that create a barrier to HIV eradication [3–6]. Early infiltration of gut tissues is facilitated by the local abundance of CD4+/C-C chemokine receptor type 5 (CCR5)+ T cells that are targeted for infection by the virus [7–10] and potentially by interaction between the HIV envelope and gut-homing integrin receptors such as α4β7, although the clinical significance of this interaction remains unclear [11]. This reservoir of infection persists in patients who begin antiretroviral therapy (ART) during the chronic phase of HIV infection, who often still demonstrate detectable proviral HIV DNA [12–17], HIV RNA [15–20] and replication-competent virus [21] in the gut despite years of treatment that suppresses the virus in the periphery.

Acute HIV infection (AHI) is associated with a surge in peripheral blood cellular and inflammatory biomarkers such as alpha interferon (IFN-α), C-X-C motif chemokine ligand 10 (CXCL10; also known as interferon gamma-induced protein 10), tumour necrosis factor alpha (TNF-α), monocyte chemotactic protein 1 (MCP-1) and CD8+ T cell activation [22–28]. Studies have shown that ART initiation during acute or early HIV infection reduces inflammation, reduces HIV reservoir size and improves immune reconstitution in the peripheral blood [29–32]. However, even ART initiated during AHI fails to induce complete reconstitution of gut mucosal immunity in most patients [19,33–35].

Identifying correlates of initial gut mucosal infiltration can help improve our understanding of HIV pathogenesis and may inform efforts at viral eradication. Understanding the dynamics of HIV burden and gut mucosal immunity that surround gut mucosal infiltration could inform interventions to prevent or reverse the deleterious effects of this event. In this study, we investigate associations between markers of HIV burden, immune activation and inflammation across multiple body compartments in participants who initiated ART during AHI, stratified by whether HIV RNA was detectable in colonic mucosal biopsy specimens at the time of ART initiation. We also assessed the impact of 24 weeks of ART on these parameters.

## Methods

### Study population

The ongoing RV254/SEARCH010 cohort study (clinicaltrials.gov NCT00796146) prospectively enrols participants at the Thai Red Cross AIDS Research Centre in Bangkok. Individuals presenting for HIV testing during AHI are identified in real-time according to a previously published algorithm [36]. Briefly, samples are screened using a fourth generation (4thG) immunoassay (IA) detecting HIV antigen and HIV immunoglobulin M (IgM). Non-reactive samples undergo pooled nucleic acid testing (NAT) and reactive samples are tested using a less-sensitive second generation (2ndG) IA sensitive to HIV IgG only. Individuals are offered enrolment into the study if they have either a non-reactive 4thG IA and a positive NAT or a reactive 4thG IA and a non-reactive 2ndG IA.

Individuals are also offered initiation of ART during AHI via a separately funded protocol, as previously described [37]. All participants receive ART that includes efavirenz, tenofovir and either emtricitabine or lamivudine. The first 10 participants to enrol received intensified therapy that also included raltegravir and maraviroc. Subsequent participants were randomized in a 1:1 ratio to receive either the three-drug or five-drug regimen.

Participants who enrol in RV254/SEARCH010 undergo serial interviews, physical examinations and phlebotomy. Participants may also participate in optional procedures including leukapheresis, colon biopsy and lumbar puncture to collect cerebrospinal fluid (CSF). Participants diagnosed with AHI between May 2009 and March 2012 who underwent colon biopsy at the time of enrolment are included in this analysis.

All participants provided written informed consent prior to enrolment in the RV254/SEARCH010 cohort and separate consent for optional procedures. The study protocol was approved by institutional review boards at Chulalongkorn University, Bangkok, Thailand, and the Walter Reed Army Institute of Research, Silver Spring, MD, USA.

### Staging of acute HIV infection and determination of HIV subtype

AHI was staged using blood from the day of enrolment into the cohort according to the system described by Fiebig et al. [38]. Fiebig stages I to V were considered AHI. HIV subtype was determined using the multiregion hybridization assay [39] or HIVSeq [40] programme.

### Estimation of HIV exposure date

A detailed sexual history was obtained upon enrolment for each participant and reviewed by a committee of at least three study staff. Sexual encounters prior to diagnosis with HIV were categorized as very high risk (such as condomless sex or injection drug use), medium risk (such as anal or vaginal sex with a condom) or low risk (such as insertive oral sex or receptive oral sex without ejaculation). The estimated HIV exposure date was calculated as the mean of the dates of encounters in the highest risk category reported by each participant within 30 days prior to diagnosis. Sexual encounters up to 60 days prior to diagnosis were included in the calculation if a participant reported no sexual activity within 30 days or the participant was determined to be in Fiebig stage III or later with very high risk behaviour in the period 30 to 60 days prior to diagnosis and lower risk behaviour within 30 days. The duration since HIV exposure was calculated by subtracting the estimated HIV exposure date from the date of HIV diagnosis.

### Biopsy processing

Participants underwent a routine sigmoidoscopy procedure under moderate conscious sedation. Approximately 30 endoscopic biopsies were randomly collected from the sigmoid colon using Radial Jaw 3 biopsy forceps (Boston Scientific, Natick, MA, USA). Participants were screened for incidental histopathology using one or two of these biopsy pieces.

Flow cytometry was performed on freshly isolated colonic mucosal mononuclear cells (CMMCs) from 20 to 25 biopsy pieces that were processed within 30 minutes of collection. In groups of five, the biopsies were weighed and placed in 500 mL of Roswell Park Memorial Institute (RPMI) media containing 10% human AB serum (Gemini Bio-Product, West Sacramento, CA, USA), 1% HEPES, 1% L-glutamine, 0.1% gentamicin (Invitrogen, Carlsbad, CA, USA), 1% penicillin/streptomycin and 2.5 mg/mL amphotericin B (Invitrogen). Samples were then digested using 0.5 mg/mL Collagenase II (Sigma, St. Louis, MO, USA). After digestion, samples were filtered through a cell strainer using a syringe with a 16-gauge blunt end needle. This procedure was repeated once or twice in case undigested tissue remained. After being washed twice with RPMI containing 1% HEPES, 1% L-glutamine, 1% penicillin/streptomycin, 0.1% gentamicin and 2.5 mg/mL amphotericin B, CMMCs were counted and viable cell enumeration was determined using trypan blue exclusion and Beckman Coulter AcT 5 haematology analyzer (Fullerton, CA, USA).

One or two biopsy pieces were collected in phosphate buffered saline and subsequently stored in 1 mL of RNAlater (Ambion, Foster, CA, USA) at −80°C for HIV RNA quantification to be performed at a later time. If sufficient material was available, then biopsy pieces stored in RNA later were also used for HIV DNA quantification.

### Quantification of HIV RNA and DNA

Colonic HIV RNA was measured using one to two frozen biopsy specimens that were weighed, homogenized using a mortar and pestle and suspended in AVL buffer as provided in the QIAamp Viral RNA Mini Kit (Cat No. 52904, Qiagen NV, Hilden, Germany). Extraction was completed according to the kit instructions. HIV RNA was quantified using the Siemens Quantiplex HIV-1 3.0 assay with a lower limit of detection of 50 copies/mg (Siemens Healthcare, Erlangen, Germany). The average amount of tissue used for this assay was 5.2 mg (range 1 to 16 mg).

HIV RNA was measured in the plasma and CSF using either the Roche Amplicor HIV-1 Monitor Test v1.5 or the Roche COBAS AmpliPrep/COBAS TaqMan HIV-1 Test v2.0 (Roche Diagnostics, Branchburg, NJ, USA). In the serum, the lower limits of detection for these assays are 50 and 20 copies/mL, respectively. In the CSF, the lower limits of detection are 100 and 80 copies/mL.

Total HIV DNA quantification was performed using a modified nested PCR with primers and probes specifically designed for HIV subtypes CRF01_AE and B as previously described [30,41].

### Measurement of soluble inflammatory markers

Levels of inflammatory cytokines and chemokines, including TNF-RII, IL-6, IL-17 and MCP-1, were assayed in citrate plasma in duplicate using Luminex multiplex technology according to the manufacturer's instructions (Millipore, Billerica, MA, USA). Single-analyte ELISAs were performed to measure CXCL10 (Life Technologies, Grand Island, NY, USA), neopterin (GenWay Biotech, San Diego, CA, USA), IFN-α, I-FABP and sCD14 (R&D Systems, Minneapolis, MN, USA) and analyzed using SoftMax Pro (Molecular Devices, Sunnyvale, CA, USA). D-dimer was measured by ELISA on a VIDAS instrument (bioMerieux Inc., Durham, NC, USA), and C-reactive protein was measured by electrochemiluminescence (Meso Scale Discovery, Gaithersburg, MD, USA).

### Immunophenotyping

Immunophenotyping was performed on cryopreserved peripheral blood mononuclear cells (PBMCs) and freshly-isolated CMMCs from sigmoid colon. Cells were first stained with Aqua Live/Dead dye (Invitrogen, Eugene, OR, USA). Subsequently the activation status of CD4+ and CD8+ T cells was determined by staining cells using anti-CD3 PE-Cy7 (Invitrogen), anti-CD4 ECD (Beckman Coulter, Brea, CA, USA), anti-CD8 PerCP-Cy5.5, anti-HLA-DR V450 and anti-CD38 APC (BD Bioscience, San Jose, CA, USA) for 20 minutes at room temperature. Post staining cells were resuspended in 1% formaldehyde and acquired within 24 hours using a custom-built BD LSRII or Fortessa flow cytometer (BD, San Jose, CA, USA) and analyzed using FlowJo software version 9.6.3 or higher (TreeStar, Ashland, OR, USA). At least 80,000 live cells were acquired in the lymphocyte gate.

### Calculation of absolute numbers of colonic T cell subsets

Absolute numbers of CD4+ and CD8+ T cell subsets per gram of gut tissue were calculated by multiplying the total viable cell count per gram by percentages obtained from flow cytometry analysis. The total cell count per gram of tissue was calculated by dividing the viable cell count by the tissue weight. This proportion was then multiplied by the percent of cells in the live lymphocyte gate and that number was subsequently multiplied by the percent of CD3+ lymphocytes. The absolute number of colonic CD3+ T cells was used in conjunction with the subset percentages to determine the absolute number of each T cell subset per gram of biopsy tissue.

### Statistical analyses

Colonic HIV RNA was characterized as detectable (≥50 copies/mg) or undetectable (<50 copies/mg). Variables of interest were stratified according to colonic HIV RNA detectability before the initiation of ART. Comparisons were made using the Mann-Whitney U test for continuous variables, Fisher's exact test for categorical variables and Wilcoxon paired signed rank test to compare values before and after 24 weeks of ART. Spearman's rank correlation coefficient was calculated to evaluate correlation between baseline colonic HIV RNA as a continuous variable and various markers of HIV burden and immune activation across multiple compartments. If values were undetectable for any given assay, then the lower limit of detection of the assay was used for statistical analyses. A two-sided type I error of 5% was considered statistically significant for all analyses and no formal adjustment was made for multiple comparisons. Analyses were performed using GraphPad Prism 6.0 (GraphPad Software, San Diego, CA, USA) and Stata 13.0 (StataCorp LP, College Station, TX, USA).

## Results

### Study population

During the study period, 49,458 samples were prospectively screened and 74 participants were enrolled in the cohort during AHI. Of these, 41 underwent colon biopsy at the time of enrolment, including 40 who also initiated ART during AHI (Supplementary file 1). The untreated participant was included in analyses performed on baseline data but not analyses of data after 24 weeks of follow-up in the cohort. The subgroup of participants who underwent colon biopsy did not differ from the subgroup who declined this procedure in terms of age, gender, HIV risk factor, body weight, days since HIV exposure, Fiebig stage, HIV subtype or ART regimen. Colon biopsy was performed a median of two (interquartile range (IQR) 1 to 3) days after enrolment, five (IQR 4 to 6) days after HIV diagnosis and 18 (IQR 14 to 24) days after estimated HIV exposure. Colon biopsy was performed before the initiation of ART. ART was initiated a median of two (IQR 2 to 3) days after enrolment, five (IQR 4 to 6) days after HIV diagnosis and 18 (IQR 13 to 22) days after estimated HIV exposure. Thirty-four of the participants who underwent colon biopsy also underwent lumbar puncture upon enrolment. Again, participants consenting to lumbar puncture did not differ from the participants who declined this procedure by any of the captured demographic characteristics (data not shown).

Thirty-one of the 42 participants included in this analysis had detectable colonic HIV RNA at the time of enrolment and 10 had undetectable colonic HIV RNA (Table 1). The groups with detectable and undetectable colonic HIV RNA were similar in age (median 29 and 28 years, respectively), gender (94 and 90% male, respectively) and weight (median 61 kg in both groups). Participants with detectable HIV RNA tended to be in a later Fiebig stage compared to participants in the undetectable group (6 and 60% Fiebig I, respectively, *p<*0.01) and had a longer reported duration since HIV exposure (median 16 and 11 days, respectively, *p=*0.02).

**Table 1:** Study population characteristics at enrolment

	Colonic HIV RNA	
	
Characteristics	Detectable (*n=*31)	Undetectable (*n=*10)
Age, median (IQR)	29 (24 to 32)	28 (25 to 42)
Male, *n* (%)	29 (94)	9 (90)
Risk group, *n* (%)		
MSM	29 (94)	9 (90)
Heterosexual female	2 (6)	1 (10)
Body weight (kg), median (IQR)	61 (56 to 68)	61 (56 to 67)
Days since HIV exposure, median (IQR)	16 (13 to 22)	11 (8 to 16)
Fiebig stage, *n* (%)		
I	2 (6)	6 (60)
II	7 (23)	3 (30)
III	17 (55)	1 (10)
IV	2 (6)	–
V	3 (10)	–
HIV subtype		
CRF01_AE	26 (84)	8 (80)
B	2 (6)	1 (10)
CRF01_AE/B	3 (10)	1 (10)
Antiretroviral therapy		
TDF/XTC/EFV	8 (26)	4 (40)
TDF/XTC/EFV/RAL/MVC	22 (71)	6 (60)
None	1 (3)	–

EFV, efavirenz; IQR, interquartile range; MSM, men who have sex with men; MVC, maraviroc; RAL, raltegravir; TDF, tenofovir disoproxil fumarate; XTC, lamivudine (3TC) or emtricitabine (FTC).

### Primary analysis

**Figure 1:**
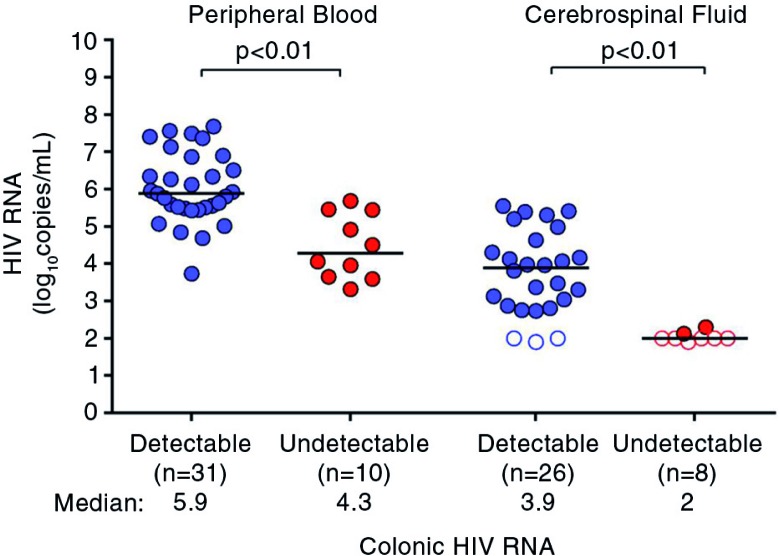
HIV RNA in the peripheral blood and cerebrospinal fluid during acute HIV infection. HIV RNA measurements during acute HIV infection are compared between participants with detectable colonic HIV RNA and undetectable colonic HIV RNA. Statistically significant pairwise comparisons (*p*<0.05) are identified. Open circles indicate values below the limit of detection.

At baseline, HIV RNA was higher in the peripheral blood and CSF in the group with detectable colonic HIV RNA as compared to the undetectable group (Figure 1). After 24 weeks of ART, peripheral blood HIV RNA was suppressed to undetectable in all participants except for one in the detectable group (2.2 log_10_ copies/mL). Similarly, colonic HIV RNA was undetectable for most participants after ART, except for two in the initially detectable group (2.4 and 3.0 log_10_ copies/mg); peripheral blood HIV RNA was undetectable in both of these participants (data not shown).

**Figure 2:**
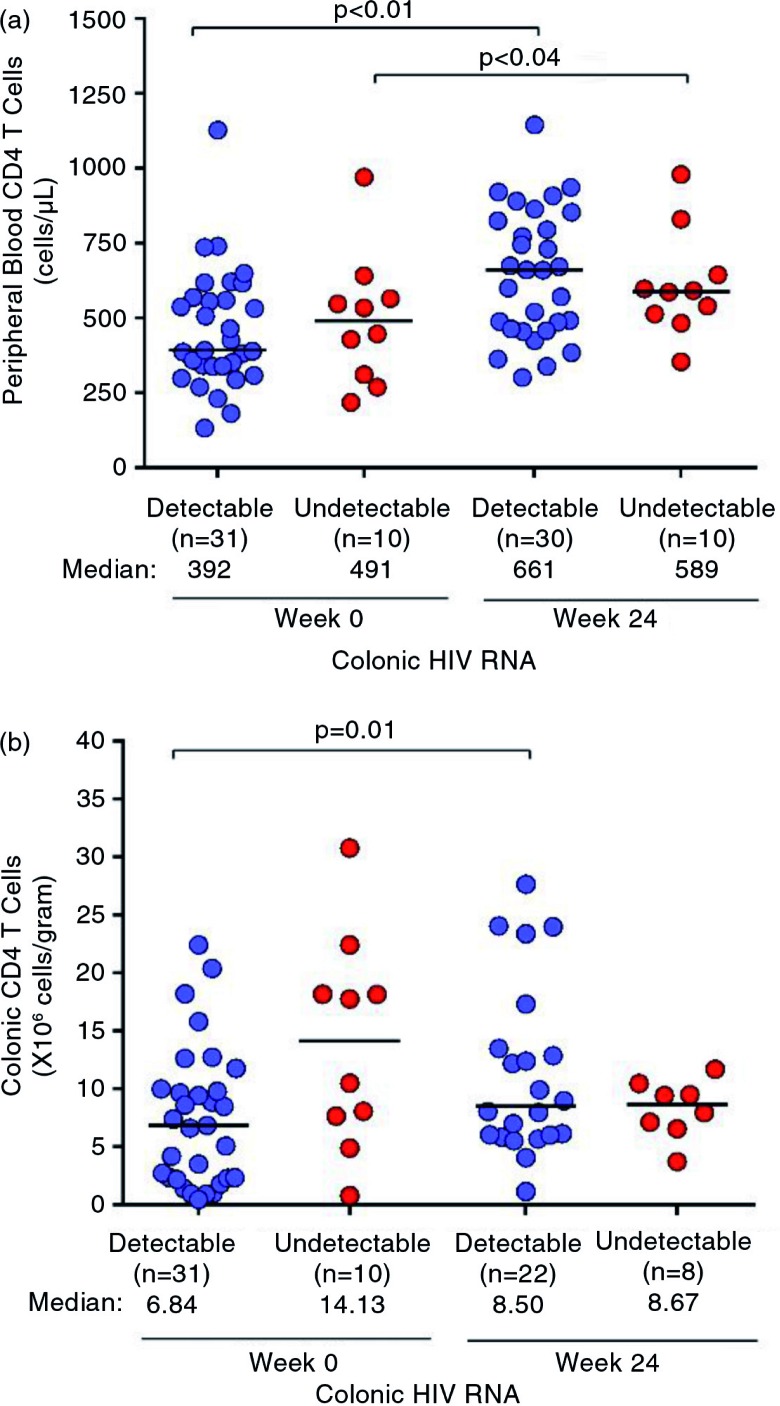
CD4 count in the peripheral blood and colon before and after ART. Absolute CD4 count measurements in the (a) peripheral blood and (b) colon are compared before and after 24 weeks of ART. Statistically significant pairwise comparisons (*p*<0.05) are identified. ART, antiretroviral therapy.

At baseline, peripheral blood CD4 count did not differ significantly between the groups with detectable and undetectable colonic HIV RNA (median (IQR) 392 (338 to 569) vs. 491 (311 to 565) cells/mm^3^, *p=*0.70, Figure 2a). There was a trend towards lower colonic CD4 count in the group with detectable colonic HIV RNA (6.8 (2.3 to 10.0) vs. 14.1 (7.6 to 18.2)×10^6^ cells/gram of tissue, *p=*0.06, Figure 2b). After 24 weeks of ART, no differences in peripheral blood or colonic CD4 counts were observed between the detectable and undetectable groups. Colonic CD4 count increased in the group with detectable colonic HIV RNA at baseline (6.8 (2.3 to 10.0) vs. 8.5 (6.0=13.5)×10^6^ cells/gram, *p=*0.01), whereas a numeric decline in the undetectable group did not achieve statistical significance (14.1 (7.6 to 18.2) vs. 8.7 (6.8 to 10.0)×10^6^ cells/gram, *p=*0.40).

**Figure 3:**
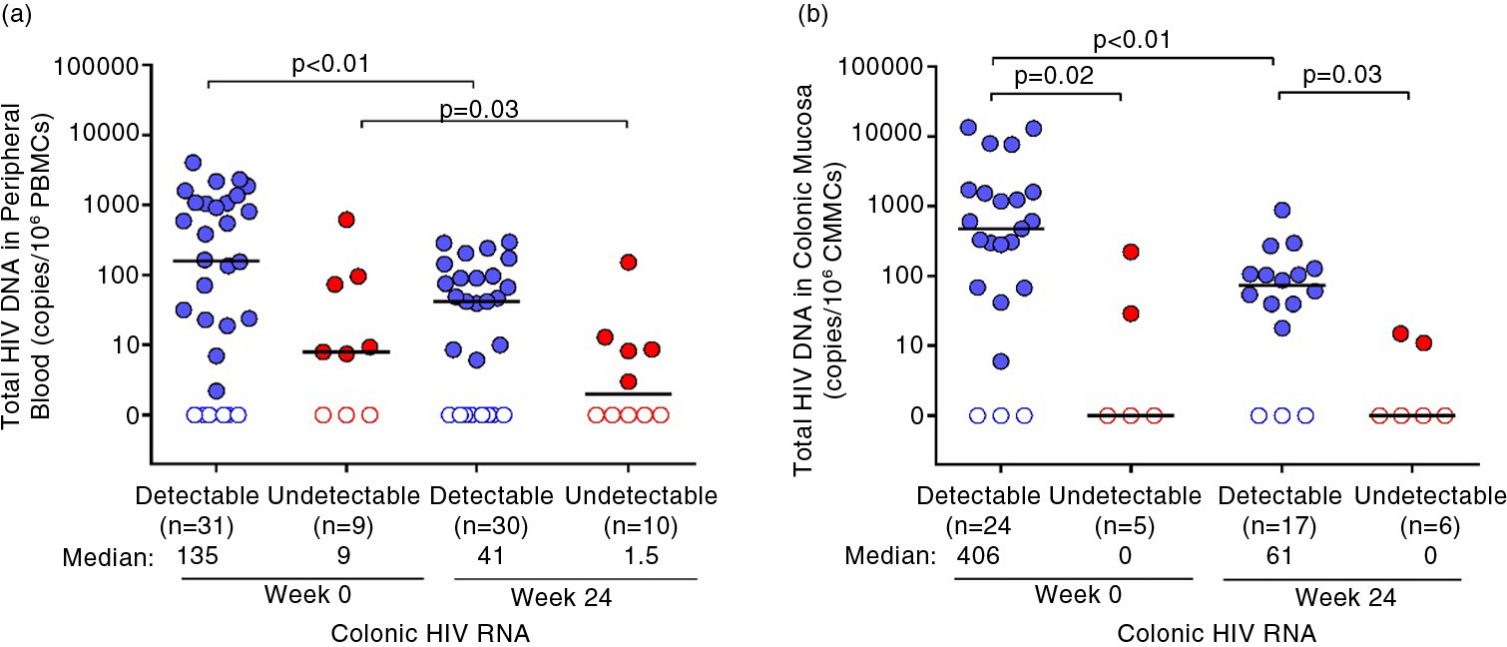
Total HIV DNA in the peripheral blood and colon before and after ART. Total HIV DNA measurements in the (a) peripheral blood and (b) colon are compared before and after 24 weeks of ART. Statistically significant pairwise comparisons (*p*<0.05) are identified. Open circles indicate values below the limit of detection. ART, antiretroviral therapy.

At baseline, total HIV DNA in CMMCs was higher in the detectable group than in the undetectable group (406 (55 to 1663) vs. 0 (0 to 29) copies/10^6^ CMMCs, *p=*0.02, Figure 3b). After 24 weeks of ART, total HIV DNA in CMMCs decreased in both groups, but remained higher in the group that had detectable colonic HIV RNA at baseline (61 (0 to 107) vs. 0 (0 to 11) copies/10^6^ CMMCs, *p=*0.02, Figure 3b). There was a similar trend towards higher total HIV DNA in PBMCs in the detectable group than in the undetectable group (135 (2 to 1050) vs. 9 (8 to 74) copies/10^6^ PBMCs, *p=*0.10). Total HIV DNA in PBMCs decreased in both groups after 24 weeks of ART, though this decrease did not achieve statistical significance (41 (0 to 91) vs. 1.5 (0 to 9) copies/10^6^ PBMCs, *p=*0.06, Figure 3a).

**Figure 4:**
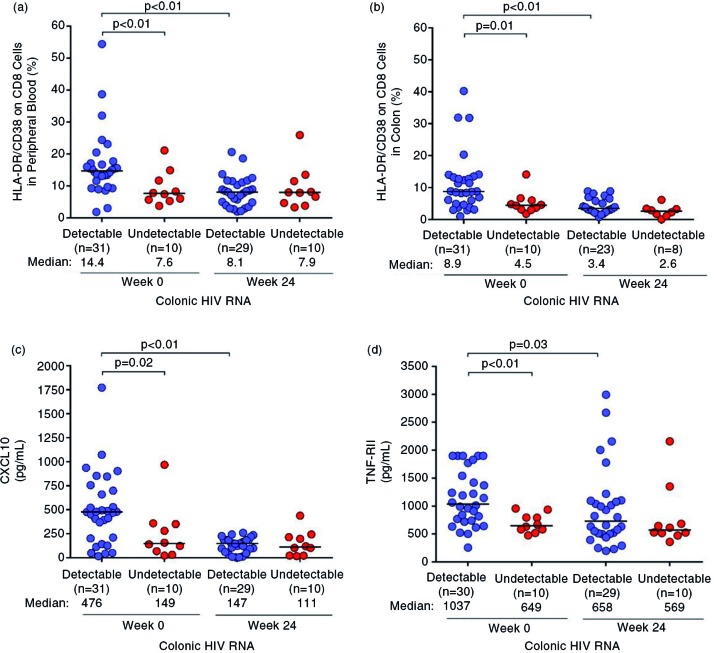
Immune activation and inflammation before and after ART. Markers of immune activation and inflammation are compared before and after 24 weeks of ART, including (a) CD8 activation in the peripheral blood, (b) CD8 activation in the colon, (c) CXCL10 and (d) TNF-RII. Statistically significant pairwise comparisons (*p*<0.05) are identified. ART, antiretroviral therapy.

CD8+ T cell activation was higher among those with detectable colonic HIV RNA at baseline in both the peripheral blood (14.4% (9.7 to 17.1%) vs. 7.6% (5.7 to 11.7%), *p*<0.01, Figure 4a) and colon (8.9% (4.9 to 13.5%) vs. 4.5% (3.2 to 6.0%), *p=*0.01, Figure 4b) but this difference disappeared after 24 weeks of ART. Similar trends were observed among soluble inflammatory markers such as CXCL10 (baseline 476 (201 to 698) vs. 149 (68 to 351) pg/mL, *p=*0.02, Figure 4c), neopterin (baseline 2405 (1743 to 3196) vs. 1368 (866 to 1910) pg/mL, *p=*0.01) and TNF-RII (1037 (739 to 1543) vs. 649 (581 to 793) pg/mL, *p*<0.01, Figure 4d).

### Sensitivity analyses

In a *post hoc* sensitivity analysis, the study population was limited to the 18 participants in Fiebig stages I and II in order to minimize differences in disease characteristics other than colonic infiltration between the two groups of interest to this study. Statistically significant baseline differences in peripheral blood and CSF HIV RNA, peripheral blood and colonic CD4 count, and total HIV DNA in PBMCs and CMMCs were no longer apparent in the more limited study population (Supplementary file 2).

There was a non-significant numerical increase in CD8+ T cell activation in the group with detectable colonic HIV RNA as compared to the undetectable group (HLA-DR/CD38 expression 9.69% (7.45 to 16%) vs. 7.43% (5.7 to 8.26%), *p=*0.20). A similar non-significant increase was observed for baseline CXCL10 (479 (201 to 854) vs. 140 (68 to 281) pg/mL, *p=*0.14). There was a trend towards higher total HIV DNA in the colon after 24 weeks of ART in the group with initially detectable colonic HIV RNA (96 (43.5 to 201.5) vs. 0 (0 to 0), *p=*0.06).

### Correlation analysis

Colonic HIV RNA, measured as a continuous variable, correlated with HIV RNA in the peripheral blood (Spearman's coefficient (*r*_s_)=0.48, *p*<0.01) and CSF (*r*_s_=0.52, *p*≤0.01). A direct correlation was also observed with peripheral CD8+ T cell activation as measured by HLA-DR/CD38 expression (*r*_s_=0.44, *p<*0.01) but not colonic CD8+ T cell activation (*r*_s_=0.18, *p=*0.26). Associations between colonic HIV infiltration and peripheral inflammatory markers that were significant in the primary analysis were not reflected in the correlation analysis of markers such as CXCL10 (*r*_s_=0.18, *p=*0.25), neopterin (*r*_s_=0.30, *p=*0.06) and TNF-RII (*r*_s_=0.19, *p=*0.24). A positive correlation was observed between colonic HIV RNA and total HIV DNA in CMMCs (*r*_s_=0.62, *p<*0.01).

## Discussion

The results of this study highlight the rapidity and breadth of viral infiltration during AHI. Detectable colonic HIV RNA is common soon after HIV infection and correlates with increased HIV burden across multiple body compartments. Participants with detectable colonic HIV RNA during AHI demonstrate colonic CD4+ T cell depletion, peripheral inflammation and CD8+ T cell activation in both colon and periphery. Early initiation of ART largely reverses these potentially harmful characteristics.

The majority of participants in this study had detectable colonic HIV RNA after very recent acquisition of HIV infection. This is consistent with other studies of HIV and non-human primate models indicating that the gut mucosa is one of the first sites infiltrated by HIV [1,2,42,43]. The direct correlation between colonic and peripheral HIV RNA levels suggests that measurement of the latter, which is much more readily performed, may be a useful surrogate marker for the burden of colonic HIV infiltration during AHI. Furthermore, interventions designed to prevent colonic infiltration, such as anti-α4β7 monoclonal antibody administration to block interaction between the HIV envelope and gut-homing integrins, may best be conducted before peripheral blood HIV RNA reaches peak levels [44]. A strong correlation was also observed between colonic and CSF HIV RNA, even when restricting the study population to participants in Fiebig stages I and II. This raises the possibility that interventions optimized for prevention of colonic infiltration by HIV may also prevent or attenuate sequelae in other body compartments, such as the central nervous system.

Participants with detectable colonic HIV RNA tended to have lower absolute CD4 counts in the colon as compared to participants with undetectable colonic HIV RNA. CD4+ T cell depletion, alongside direct effects of HIV on the colonic mucosa, disrupts mucosal integrity and enables microbial translocation, which is associated with immune activation and inflammation [45]. This pathway could explain the association observed in this study between colonic infiltration by HIV and markers of peripheral inflammation. Gut mucosal CD4+ T cell depletion has previously been shown to directly correlate with both local and peripheral CD8+ T cell activation during AHI [46].

After 24 weeks of ART, many baseline differences between the two groups in this study disappear. This suggests that even if colonic infiltration by HIV has already occurred, much of the unfavourable phenotype associated with that event can be reversed with early ART. ART initiated during chronic HIV infection often fails to restore mucosal immunity and T cell homeostasis in the GALT [19,20,47], which may drive ongoing inflammation and immune activation [19,35,48,49]. Reduction of inflammation is presumably desirable, as chronic inflammation in the setting of HIV infection has been linked to complications such as cardiovascular disease, opportunistic infections, neurologic disorders and non-AIDS-defining events [50–55]. CD8+ T cell activation decreased in the group that started ART after colonic HIV infiltration so that no difference between the two groups was detectable after 24 weeks of ART. However, CD8+ T cell activation has been shown to be more robust in HIV non-progressors [56], and the magnitude of CD8+ T cell response during acute infection is inversely correlated with viral set point [57], suggesting that activation of these cells may be an important component of efforts to achieve HIV remission off ART. Total HIV DNA remained higher after 24 weeks of therapy in both the peripheral blood and colon of participants who started ART after colonic infiltration, underscoring the difficulty of eradicating the viral reservoir once integration into the host genome has occurred. Low levels of PBMC-associated HIV DNA were associated with post-treatment control in both the VISCONTI [58] and SPARTAC [59] studies. The optimal timing of ART initiation to maximize the likelihood of post-treatment control remains uncertain, as this must balance seemingly opposing goals of both a small HIV reservoir and potent HIV-specific immune responses [60,61].

This study utilized a unique and well-characterized cohort of individuals who initiated ART during AHI and agreed to invasive procedures to characterize HIV burden and biomarkers across multiple body compartments. Although all participants underwent baseline colon biopsy, the analysis is limited by smaller sample sizes for other specimens and time points. The analysis includes no untreated comparator group, so comparisons cannot be drawn between markers of inflammation or immune activation among participants in this study as compared to an HIV-uninfected Thai population. Findings from this cohort may not be generalizable to other populations with epidemics caused by other clades of virus or in populations other than men who have sex with men, such as those exposed to HIV via the vaginal or intravenous route. This analysis is also limited by variability in sampling of the colonic mucosa, which is a large surface that may not be completely characterized by a small number of random biopsies. Colonic HIV RNA measurements were normalized by volume, but not to any housekeeping genes. HIV burden and markers of immune activation are not distributed homogenously throughout the colonic mucosa and are known to vary across different sections of the small and large bowel [62]. Lastly, it is possible that persistent differences in total HIV DNA in the colon after 24 weeks of ART may resolve with a longer duration of therapy, and additional follow-up is warranted.

## Conclusions

This study demonstrates that viral infiltration of the colon is common during even the earliest stages of AHI. Detectable HIV RNA in the colon is associated with depletion of colonic CD4 cells, systemic inflammation and increased HIV burden across other body compartments, and CD8+ T cell activation in the blood and colon. After 24 weeks of ART, many of these differences disappear, but persistent elevation of total HIV DNA in the group that experienced colonic infiltration prior to ART initiation suggests that blocking initial colonic infiltration may be a useful strategy to reduce viral reservoirs, thereby facilitating eventual viral eradication or induction of HIV remission.

## Supplementary Material

Initiation of antiretroviral therapy before detection of colonic infiltration by HIV reduces viral reservoirs, inflammation and immune activation

Initiation of antiretroviral therapy before detection of colonic infiltration by HIV reduces viral reservoirs, inflammation and immune activation

## Prior presentation

This work was presented, in part, at the 22nd Conference on Retroviruses and Opportunistic Infections, Seattle, WA, USA, 23 to 26 February 2015.
