# Supplementary material for: Initiation of antiretroviral therapy before detection of colonic infiltration by HIV reduces viral reservoirs, inflammation and immune activation
Source: J Int AIDS Soc. 2016 Sep 15;19(1):21163. doi: 10.7448/IAS.19.1.21163 (PMC5026729; doi:10.7448/IAS.19.1.21163)
Supplement: Initiation of antiretroviral therapy before detection of colonic infiltration by HIV reduces viral reservoirs, inflammation and immune activation [file JIAS-19-21163-s001.pdf]

Additional File 1
Participant Flow Diagram

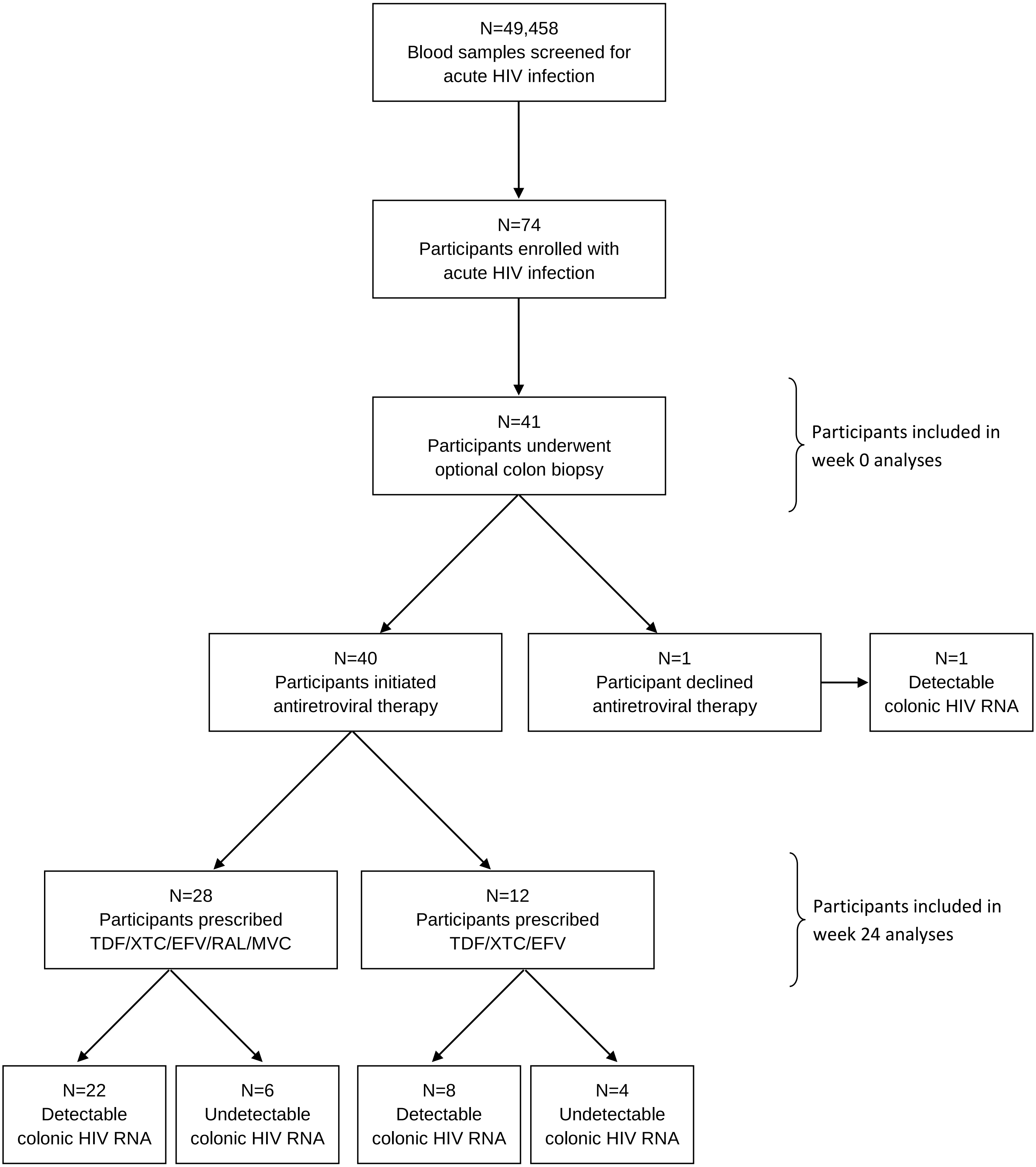

Abbreviations: TDF, tenofovir disoproxil fumarate; XTC, lamivudine (3TC) or emtricitabine (FTC); EFV, efavirenz; RAL, raltegravir; MVC, maraviroc.
