# Supplementary material for: Initiation of antiretroviral therapy before detection of colonic infiltration by HIV reduces viral reservoirs, inflammation and immune activation
Source: J Int AIDS Soc. 2016 Sep 15;19(1):21163. doi: 10.7448/IAS.19.1.21163 (PMC5026729; doi:10.7448/IAS.19.1.21163)
Supplement: Initiation of antiretroviral therapy before detection of colonic infiltration by HIV reduces viral reservoirs, inflammation and immune activation [file JIAS-19-21163-s002.pdf]

Additional File 2
Sensitivity Analysis

Table. Baseline Study Population Characteristics of Participants Included in Sensitivity Analysis

Characteristics	Colonic HIV RNA Detectable (n=9)	Colonic HIV RNA Undetectable (n=9)
**Age**, median (IQR)	30 (25 – 37)	29 (25 – 42)
**Male**, n (%)	9 (100)	8 (89)
**Risk group,** n (%)		
MSM	9 (100)	8 (89)
Heterosexual female	-	1 (11)
**Body weight** (kg), median (IQR)	61 (55 – 66)	62 (56 – 67)
**Days since HIV exposure**, median (IQR)	14 (14 – 15)	9 (8 – 14)
**Fiebig stage**, n (%)		
I	2 (6)	6 (60)
II	7 (23)	3 (30)
**HIV subtype**		
CRF01_AE	8 (89)	8 (89)
B	-	1 (11)
CRF01_AE/B	1 (11)	-
**Antiretroviral therapy**		
TDF/XTC/EFV	3 (33)	4 (44)
TDF/XTC/EFV/RAL/MVC	5 (56)	5 (56)
None	1 (11)	-

Abbreviations: IQR, interquartile range; MSM, men who have sex with men; TDF, tenofovir disoproxil fumarate; XTC, lamivudine (3TC) or emtricitabine (FTC); EFV, efavirenz; RAL, raltegravir; MVC, maraviroc.

Table. Sensitivity Analysis of Findings Before and After 24 Weeks of ART in Participants with Detectable and Undetectable Colonic HIV RNA during Fiebig Stages I/II

	Colonic HIV RNA				
	Detectable		Undetectable		
	n	Median (IQR)	n	Median (IQR)	P-value
**HIV RNA in Blood**					
Week 0	9	5.4 (5 - 6.3)	9	4.1 (3.6 - 5.4)	**0.05**
Week 24	8	1.7 (1.7 - 1.7)	9	1.7 (1.7 - 1.7)	1.00
Change from baseline		**P=0.01**		**P<0.01**	
**HIV RNA in CSF**					
Week 0	7	2.8 (2 - 4.1)	7	2 (2 - 2.1)	0.18
Week 24	3	1.9 (1.9 - 2)	4	2 (2 - 2)	0.31
Change from baseline		P=0.11		P=0.16	
**CD4 in Blood**					
Week 0	9	555 (426 - 618)	9	534 (311 - 565)	0.43
Week 24	8	783 (672.5 - 913)	9	591 (513 - 644)	0.10
Change from baseline		P=0.09		P=0.07	
**CD4 in Colon**					
Week 0	9	9.6 (4.2 - 11.8)	9	17.8 (8.1 - 18.2)	0.23
Week 24	6	9.4 (5.7 - 24)	7	7.9 (6.5 - 10.5)	0.67
Change from baseline		P=0.60		P=0.31	
**Total HIV DNA in PBMCs**					
Week 0	9	12 (0 - 32)	8	8.5 (4 - 43)	0.88
Week 24	8	3.5 (0 - 124.5)	9	0 (0 - 8)	0.34
Change from baseline		P=0.19		P=0.06	
**Total HIV DNA in CMMCs**					
Week 0	7	477 (0 - 3309)	4	0 (0 - 111)	0.11
Week 24	4	96 (43.5 - 201.5)	5	0 (0 - 0)	0.06
Change from baseline		P=0.11		P=0.32	
**HLA-DR/CD38 on CD8 cells in PBMCs**					
Week 0	9	9.7 (7.5 - 16)	9	7.4 (5.7 - 8.3)	0.20
Week 24	8	8.5 (4.8 - 11.7)	9	7.9 (4.7 - 11.5)	0.92
Change from baseline		P=0.12		P=0.48	
**HLA-DR/CD38 on CD8 cells in CMMCs**					
Week 0	9	4.6 (3 - 6.1)	9	4.3 (3.2 - 6)	0.82
Week 24	7	2.7 (1.8 - 3.4)	7	2.3 (1.1 - 3.4)	0.75
Change from baseline		P=0.06		P=0.13	
**IP-10**					
Week 0	9	479 (201 - 854)	9	140 (68 - 281)	0.14
Week 24	8	153.3 (91 - 184.3)	9	119 (23 - 214)	0.92
Change from baseline		**P=0.05**		P=0.31	
**Neopterin**					
Week 0	9	1922 (1234 - 2688)	9	1412 (866 - 1910)	0.31
Week 24	8	1405 (973 - 1811)	9	1597 (1129 - 1964)	0.50
Change from baseline		**P=0.04**		P=0.44	
**TNF-RII**					
Week 0	9	911 (641 - 1420)	9	669 (581 - 793)	0.10
Week 24	8	632 (564 - 729)	9	526 (517 - 644)	0.29
Change from baseline		P=0.40		P=0.86	

	Colonic HIV RNA				
	Detectable		Undetectable		
	n	Median (IQR)	n	Median (IQR)	P-value
**I-FABP**					
Week 0	9	1036 (266 - 1177)	9	889 (314 - 1070)	0.89
Week 24	8	2042 (989 - 4462)	9	1837 (1321 - 5786)	0.85
Change from baseline		**P=0.02**		**P=0.01**	
**sCD14**					
Week 0	9	1.3 (1.1 - 1.7)	9	1.2 (1.2 - 1.8)	0.90
Week 24	8	1 (1 - 1.2)	9	1.4 (1.3 - 1.4)	0.05
Change from baseline		P=0.09		P=0.95	
**D-Dimer**					
Week 0	9	278 (175 - 404)	9	199 (182 - 357)	0.69
Week 24	8	151 (116 - 282)	9	256 (171.4 - 301)	0.18
Change from baseline		P=0.12		P=0.86	
**C-Reactive Protein**					
Week 0	9	1.1 (0.7 - 1.3)	9	0.8 (0.4 - 1.4)	0.63
Week 24	8	0.3 (0.2 - 0.7)	9	0.6 (0.5 - 0.8)	0.08
Change from baseline		**P=0.04**		P=0.95	

Abbreviations: IQR, interquartile range; CSF, cerebrospinal fluid; PBMCs, peripheral blood mononuclear cells; CMMCs, colonic mucosal mononuclear cells. In this post-hoc sensitivity analysis, the study population was limited only to participants in Fiebig stages I and II (9 participants in each group) in order to minimize differences in disease characteristics other than colonic infiltration between the two groups of interest to this study. P-values for each row reflect comparisons between participants with detectable and undetectable colonic HIV RNA. P-values for each column reflect comparisons between assessments at week 0 (ART initiation) and after 24 weeks of ART. Statistically significant p-values are in **bold**.
